# Cardiovascular biomarker phenotype associated with immune checkpoint inhibitor therapy in patients with non-small cell lung cancer: an exploratory blood transcriptome study

**DOI:** 10.3389/fimmu.2026.1972440

**Published:** 2026-10-21

**Authors:** Joanna Ziemska-Legięcka, Natalia Żeber-Lubecka, Magdalena Borucka, Dorota Kaczmarska, Magdalena Zaborowska-Szmit, Patrycja Ozdowska, Katarzyna Zajda, Anna Janowicz-Żebrowska, Michalina Dąbrowska, Lukasz Serementa, Tadeusz M. Orlowski, Maciej Krzakowski, Sebastian Szmit, Michał Mikula, Jerzy Ostrowski, Beata Jagielska

**Affiliations:** 1 Department of Experimental Oncology, Laboratory of Cancer Metabolism, Maria Sklodowska-Curie National Research Institute of Oncology, Warsaw, Poland; 2 Department of Gastroenterology, Hepatology and Clinical Oncology, Center of Postgraduate Medical Education, Warsaw, Poland; 3 Department of Lung Cancer and Thoracic Tumours, Maria Skłodowska-Curie National Research Institute of Oncology, Warsaw, Poland; 4 Cardio-Oncology Unit, Maria Skłodowska-Curie National Research Institute of Oncology, Warsaw, Poland; 5 Department of Surgery, Department of Thoracic Surgery, National Research Institute of Chest Diseases, Warsaw, Poland; 6 Department of Cardio-Oncology, Centre of Postgraduate Medical Education, Warsaw, Poland; 7 Maria Skłodowska-Curie National Research Institute of Oncology, Warsaw, Poland

**Keywords:** blood transcriptomics, cardiovascular biomarkers, immune checkpoint inhibitors, immunotherapy, non-small cell lung cancer (NSCLC), NT-ProBNP, RNA sequencing

## Abstract

**Background:**

Immune checkpoint inhibitors (ICIs) have transformed the management of advanced non-small cell lung cancer (NSCLC), but their use is associated with potentially serious immune-related cardiotoxicity, often reflected by elevations in N-terminal pro-B-type natriuretic peptide (NT-proBNP). Identifying patients at risk before treatment initiation remains a major clinical challenge. The aim of this study was to investigate whether peripheral blood transcriptomic profiles are associated with susceptibility to an ICI-related cardiac biomarker-defined phenotype.

**Methods:**

Peripheral blood RNA sequencing was performed in patients with advanced NSCLC receiving anti-PD-1/PD-L1 therapy (n=61) and in a control group with predominantly early-stage disease (n=29). Differential expression and functional enrichment analyses were used to characterize global and baseline transcriptomic differences, early treatment-related changes, and molecular signatures associated with NT-proBNP elevation and survival.

**Results:**

Despite substantial transcriptomic overlap (86.0% of genes shared), global analysis revealed significant differences between early- and advanced-stage disease, as demonstrated by PCA and PERMANOVA. Baseline comparison in patients who subsequently developed NT-proBNP elevation identified a small set of differentially expressed genes (n=9), indicating subtle pre-treatment differences in regulatory and signaling pathways. Early response analysis demonstrated modest transcriptional changes after the first ICI cycle, with enrichment analyses pointing to immune-related pathways, including leukocyte migration and cytokine signaling. NT-proBNP-defined phenotype was associated with exploratory differential gene expression patterns and enrichment of pathways related to T-cell activation and immune signaling. Exploratory survival-associated transcriptomic profiles were characterized by enrichment of pathways related to platelet activation, coagulation and cell adhesion.

**Conclusion:**

Peripheral blood transcriptomic profiling reveals stage-dependent and clinically relevant transcriptomic patterns in NSCLC, including early immune-related transcriptional changes and signatures associated with a cardiovascular biomarker phenotype defined by NT-proBNP changes. Although these alterations are subtle, they highlight the potential of peripheral blood transcriptomic profiling to identify transcriptomic signatures associated with an NT-proBNP-defined cardiovascular biomarker phenotype. Given the exploratory nature of the analyses and the relatively small size of several subgroups, particularly the survival comparison, these findings should be interpreted as hypothesis-generating and require validation in independent cohorts.

## 1 Introduction

Blood transcriptomics, which analyzes RNA in circulating blood cells, offers a dynamic picture of an individual’s physiological state, immune response, and disease progression. Unlike static genome sequencing, blood transcriptomes reflect the influence of both genetic predisposition and environmental factors, making them a valuable tool for diagnosis, prognosis, and treatment monitoring (1). Blood transcriptomics can distinguish bacterial from viral infections, and in studies of SARS-CoV-2, transcriptomic profiling helped identify differences in the immune response between variants of varying severity (2–5). It also enables the distinction between active tuberculosis and latent infection (6, 7). RNA sequencing from blood allows the identification of abnormal splicing, gene expression outliers, or allele-specific expression, aiding the diagnosis of approximately 7% of rare genetic diseases previously undiagnosed by DNA sequencing (8, 9). Blood transcriptome signatures have demonstrated high accuracy in diagnosing sepsis and nosocomial infections in intensive care unit patients (10, 11), in monitoring autoimmune diseases (such as systemic lupus erythematosus (SLE) and inflammatory bowel diseases) and non-alcoholic fatty liver disease (12–15), and have also been able to detect tumor signatures in blood, offering the potential for early detection of several cancer types (16, 17). Studies show that a sequencing depth of 10 million reads is often sufficient to perform robust immune deconvolution (estimation of cell type abundance) in a clinical setting (18). Blood transcriptomes are sensitive to factors such as medications, diet, stress, and comorbidities (19, 20). Peripheral blood cells express a substantial proportion of genes shared across tissues, and changes in their transcriptional programs may indirectly reflect pathological processes occurring in distant inaccessible organs, particularly those driven by immune and inflammatory mechanisms. Blood sampling is minimally invasive and easy to perform multiple collections, making it ideal for monitoring chronic diseases. It is sensitive to early disease stages and subtle molecular changes before overt symptoms develop. Although technical batch effects in sample preparation can influence results, current research suggests that integrating blood transcriptomics with other “omics” data and clinical information can lead to more personalized and efficient diagnostic tools.

Previous studies demonstrated that blood-derived transcriptomic profiles, including analyses performed in Peripheral Blood Mononuclear Cells (PBMCs), showed similarity to heart tissue gene-expression profiles during toxic reactions (21). Blood transcriptomics allowed to study toxicity from tyrosine kinase inhibitors and anthracyclines and identified key mechanisms involved in cardiotoxicity, including oxidative stress, mitochondrial dysfunction, altered contractile protein expression, and extracellular matrix remodeling (21, 22). While combined with other models (such as human-induced pluripotent stem cell-derived cardiomyocytes), it provided high-sensitivity prediction of structural toxicity (23).

Peripheral Blood Mononuclear Cell (PBMC) transcriptomes showed high similarity to heart tissue gene expression profiles during toxic reactions (21). Blood transcriptomics allowed to study toxicity from tyrosine kinase inhibitors and anthracyclines and identified key mechanisms involved in cardiotoxicity, including oxidative stress, mitochondrial dysfunction, altered contractile protein expression, and extracellular matrix remodeling (21, 22). While combined with other models (such as human-induced pluripotent stem cell-derived cardiomyocytes), it provided high-sensitivity prediction of structural toxicity (23).

Blood transcriptomics differentiated patients with early-stage lung cancer from those with advanced disease, as well as from non-malignant conditions. A 29-gene signature in peripheral blood mononuclear cells (PBMCs) was identified that could distinguish non–small cell lung cancer (NSCLC) patients from controls with about 86% accuracy (24). Peripheral blood transcriptomic and machine learning algorithms demonstrated high accuracy for early lung cancer diagnosis, yielding a mean AUC of 0.938 (25). Moreover, although the exploratory analyses of microarray gene expression data in peripheral blood focusing on time-dependent processes in lung cancer cases and their matched controls indicated small differences in gene expression during the years prior to diagnosis, case–control differences were most distinguishable in metastatic case–control pairs in the period 0–3 years prior to diagnosis (26).

We hypothesized that patients with NSCLC may exhibit baseline immune- and cardiovascular-related transcriptional programs associated with the subsequent development of an NT-proBNP-defined cardiovascular biomarker phenotype during ICI therapy, even before treatment initiation and the onset of clinical manifestations. The aim of this study was therefore to determine whether blood transcriptome signatures could be linked to susceptibility to an ICI-associated cardiovascular biomarker phenotype, defined by changes in NT-proBNP levels, and to identify the early molecular alterations associated with this phenotype in patients with NSCLC.

## 2 Results

A total of 61 patients with advanced-stage NSCLC were included in the study group, while 29 patients with predominantly early-stage disease served as the control group. Peripheral blood transcriptome sequencing was performed for all samples.

To investigate transcriptional signatures associated with susceptibility to an ICI-related cardiac biomarker-based phenotype and early molecular changes associated with it, we performed a series of differential expression analyses focusing on baseline differences, treatment-induced changes, and clinically defined cardiac biomarker-defined phenotype.

### 2.1 Global transcriptomic differences between advanced and early-stage NSCLC

To characterize global transcriptomic differences associated with disease stage and prior treatment differences, we compared peripheral blood transcriptomes between patients with advanced NSCLC and those with earlier-stage disease. Importantly, these differences should be interpreted in the context of substantial clinical heterogeneity between groups, including differences in disease stage and prior treatment exposure. As shown in the Venn diagram (**Figure 1A**, RNA-seq analysis identified transcriptomic overlap between early-stage and advanced NSCLC samples. A total of 37,200 genes (86.0%) were shared between both groups, while 2,012 genes (4.7%) were unique to early-stage NSCLC and 4,050 genes (9.4%) were specific to advanced-stage NSCLC.

**Figure 1:**
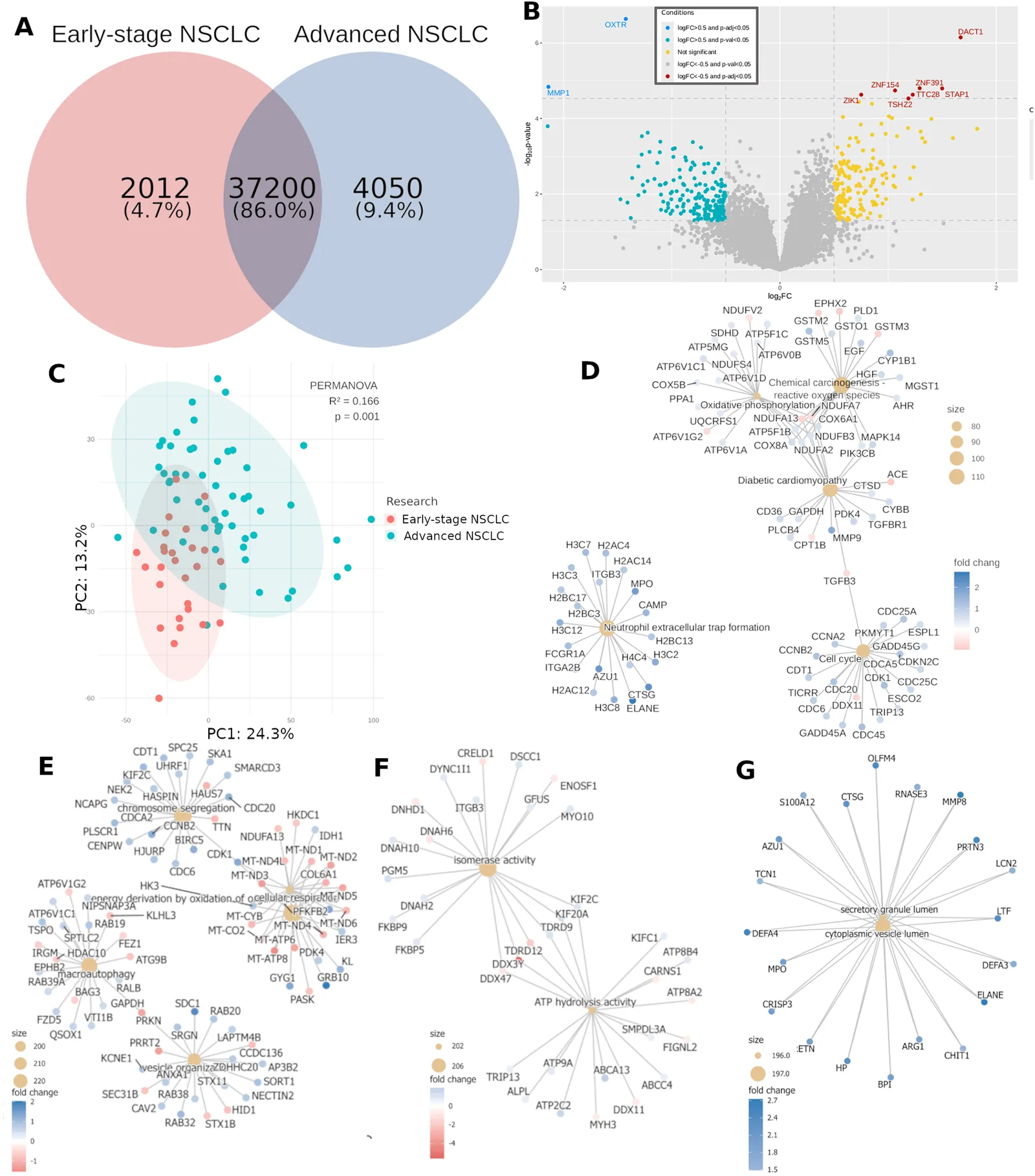
**(A)** Venn diagram showing the overlap of genes detected in early-stage and advanced NSCLC samples. A total of 37,200 genes (86.0%) were shared between both groups, while 2,012 genes (4.7%) were unique to early-stage NSCLC and 4,050 genes (9.4%) were specific to advanced-stage NSCLC. Percentages indicate the proportion of genes relative to the total number of detected genes. **(B)** Volcano plot showing all analysed genes in the comparison of baseline transcriptomic signatures. Differentially expressed genes (DEGs) are labelled at padj<0.05 and |logFC|>0.5. **(C)** Principal Component Analysis (PCA) showing differences between advanced and early-stage NSCLC. Blue dots represent the advanced NSCLC group and red dots represent the early-stage NSCLC group; ellipses represent 95% confidence intervals for each group. **(D–G)** Concept network plots presenting the top 5 most enriched terms: KEGG pathways **(D)**, biological process **(E)**, molecular function **(F)**, and cellular component **(G)**. Yellow dots represent GO terms or KEGG pathway names, with size corresponding to the number of genes included among the DEGs. Associated genes are displayed for each term or pathway and coloured according to their log(FoldChange). For visualization purposes, the number of genes per GO term was limit.

The PCA plot showed a trend toward separation between the two clinical groups based on the first two principal components (PC1: 24.3% of explained variance; PC2: 13.2%). PERMANOVA analysis confirmed a statistically significant difference between groups (R^2^ = 0.132, p = 0.001), indicating distinct overall transcriptomic profiles between early and advanced disease stages (**Figure 1C**). PCA plots for the remaining comparisons are provided in **Supplementary Figure S1**; however, no statistically significant differences between groups were observed based on PERMANOVA analysis.

In the comparison of the study group and the control group, differential gene expression analysis revealed 313 (95 protein-coding, 113 long non-coding RNA (lnc)) and 250 (201 protein-coding, 11 lncRNA) DEGs with padj<0.05, exhibiting log(FoldChange)<-1 and >1, respectively (**Supplementary Table 1**). Among the differentially expressed lncRNAs, several transcripts previously implicated in immune regulation and cancer biology were identified. These included TLR8-AS1, an antisense transcript related to innate immune signaling, as well as MEG3, LINC00861 and GAS6-AS1, which have been linked to immune regulation, cancer progression and inflammatory processes in previous studies.

Functional enrichment analysis of both unique and common protein-coding DEGs identified 137, 211, and 939 significant pathways (padj<0.05) according to the MF, CC, and BP categories of the GO database, as well as 110 KEGG pathways (**Supplementary Table 1**). The most enriched GO terms within the BP category included processes related to cellular metabolism and proliferation, such as energy derivation by oxidation of organic components, cellular respiration, chromosome segregation, and macroautophagy (**Figure 1E**). KEGG analysis further indicated enrichment of pathways associated with cell signaling and adhesion, including integrin signaling and focal adhesion (**Figure 1D**). MF analysis highlighted functions associated with enzymatic and catalytic activity, including isomerase activity, ATP hydrolysis activity, protein serine/threonine kinase activity, and GTP binding, suggesting increased metabolic and signaling activity (**Figure 1F**). CC enrichment indicated association with intracellular and membrane-related compartments, particularly vesicle and granule structures such as cytoplasmic vesicle lumen, secretory granule lumen, and other secretory vesicle components, as well as cellular structures involved in adhesion and migration, including focal adhesion and cell projection regions (**Figure 1G**).

Several enriched KEGG categories corresponded to neurodegenerative diseases, including Parkinson disease, Alzheimer disease and amyotrophic lateral sclerosis. Additional analyses demonstrated that these pathways were largely driven by genes involved in oxidative phosphorylation and mitochondrial function, which are shared across multiple KEGG disease annotations. Therefore, these pathways were not interpreted as disease-specific findings and were excluded from the final visualization to avoid overinterpretation.

### 2.2 Baseline transcriptomic signatures associated with cardiotoxicity susceptibility

To identify baseline transcriptomic features associated with susceptibility to an NT-proBNP-defined cardiovascular biomarker phenotype, we compared pre-treatment blood transcriptomes between patients who subsequently developed elevated NT-proBNP levels and those who did not which was used as a surrogate marker of cardiotoxicity; NT-proBNP is an essential diagnostic and prognostic biomarker for heart failure (27, 28).

This analysis identified nine differentially expressed genes (padj<0.05) out of 14–466 genes (**Supplementary Table 2**). Among them, genes involved in transcriptional regulation (ZNF family members), signal transduction (STAP1), and WNT pathway modulation (DACT1) were upregulated, while genes related to extracellular matrix remodeling (MMP1) and neuroendocrine signaling (OXTR) were downregulated (**Figure 1B**). These genes may indicate subtle and exploratory baseline differences in transcriptional regulation, signaling pathways, and neuro-immune interactions, which require cautious interpretation.

### 2.3 Early response to ICI

To assess early molecular responses to ICI treatment, pairwise differential analysis was performed in individuals from both subgroups, comparing the transcriptome profiles of blood collected before and after the first cycle of ICI treatment. After applying the filtering criteria, of the 16–970 genes analyzed, one gene, IL7TR, was underexpressed after the first cycle of treatment (logFC=1.05) (**Supplementary Table 3**). There were no overexpressed genes with logFC>1, but 19 DEGs exhibited logFC>0.5 fold (entrezIDs: SBNO2, MSRB1, FLOT2, ADGRE2, CXCR1, CEBPB, FPR1, CXCR2, S100A9, THBS1, HRH2, IFITM2, FPR2, BASP1, AQP9, NIBAN1, PHC2, DUSP1, TLR2) (as presented in **Figure 2A**). Although the magnitude of transcriptional changes was limited, the identified genes pointed to coordinated involvement of immune-related pathways.

**Figure 2:**
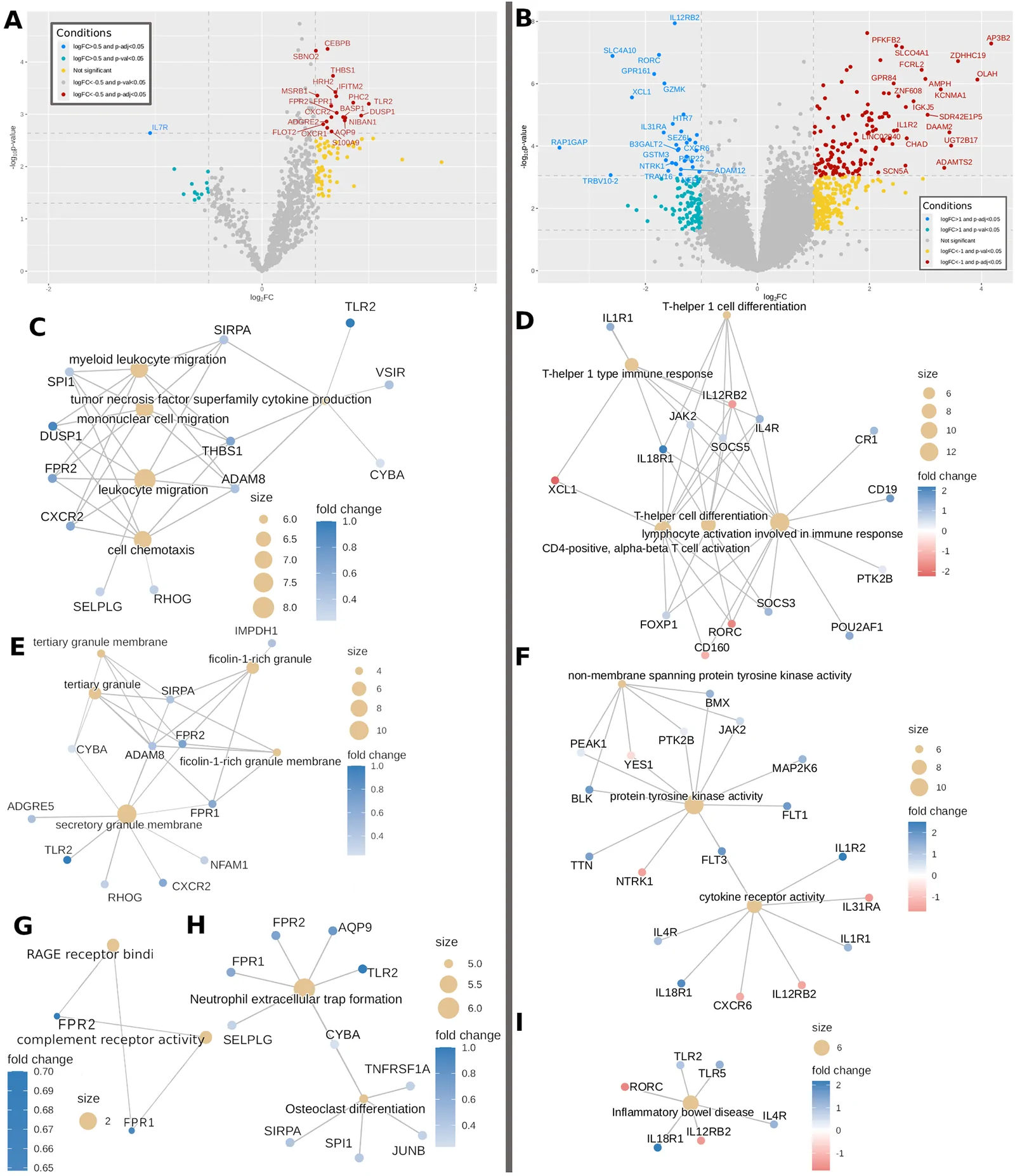
**(A)** Volcano plot showing all analysed genes in the comparison of early response to ICI; DEGs are labelled at padj<0.05 and |logFC|>0.5. **(B)** Volcano plot showing all analysed genes in the comparison of short- and long-term survival; the top 20 genes by logFC and genes with padj<0.05 and |logFC|>0.5 are labelled. **(C, E, G, H)** Concept network plots showing the top 5 enriched terms for the early-response comparison: biological process **(C)**, molecular function **(E)**, cellular component **(G)**, and KEGG pathways **(H)**. **(D, F, I)** Concept network plots showing the top 5 enriched terms for the survival comparison: biological process **(D)**, molecular function **(F)**, and KEGG pathways **(I)**. Node size indicates the number of associated DEGs, and gene colour represents log(FoldChange).

Functional enrichment analysis of DEGs identified 12, 33, and 75 significant ontology terms (padj<0.05) according to the MF, CC and BP aspects of the GO database, as well as 13 KEGG pathways (**Supplementary Table 3**). Using enrichplot in a concept network, which connected results via their overlapping genes, the most enriched ontology terms within the BP category were leukocyte migration, myeloid leukocyte migration, leukocyte chemotaxis, and cell chemotaxis (**Figure 2C**), while KEGG analysis highlighted neutrophil extracellular trap formation, cytokin-cytokine receptor interaction, and chemokine signaling pathways (**Figure 2H**). In addition, MF analysis revealed enrichment of functions related to immune receptor activity, cytokine receptor activity, cytokine binding, and RAGE receptor binding, suggesting activation of receptor-mediated immune signaling pathways (**Figure 2G**). CC analysis indicated enrichment of genes associated with membrane-associated compartments involved in immune activation and secretion, including secretory granule membrane, tertiary granule, membrane raft, and focal adhesion, pointing to the involvement of cellular structures linked to immune cell activation, signaling, and migration (**Figure 2E**).

### 2.4 Transcriptomic signatures associated with cardiac biomarker-defined phenotype

To investigate transcriptomic features associated with cardiac biomarker-defined phenotype, we stratified patients based on NT-proBNP levels after the first cycle of ICI treatment, and differential gene expression analysis of blood transcriptome profiles uncovered 32 (26 protein-coding, 2 lncRNA) and 146 (98 protein-coding, 27 lncRNA) DEGs with padj<0.05, which showed log(FoldChange)<-1 and >1, respectively (**Figure 2B**) among 16,806 genes analyzed (**Supplementary Table 4**). Among the differentially expressed lncRNAs, several transcripts previously implicated in immune regulation and cancer biology were identified, including MAILR (macrophage interferon regulatory lncRNA), MIR181A1HG, SBF2-AS1 and RALGPS2-AS1.

Functional enrichment analysis identified 3 and 15 significant GO terms (padj<0.05) according to the MF and BP ontologies, as well as a single KEGG pathway (**Supplementary Table 4**). The most significant pathways according to GO enrichment were related to lymphocyte activation involved in immune response, T-helper cell differentiation, and CD4-positive, alpha-beta T cell activation (**Figure 2D**), while KEGG analysis indicated inflammation-related signaling represented by the inflammatory bowel disease pathway (**Figure 2I**). MF analysis further highlighted enrichment of functions associated with protein tyrosine kinase activity and cytokine receptor activity, pointing to the activation of receptor-mediated signaling pathways involved in immune cell activation and intracellular signal transduction (**Figure 2F**). No significant enrichment was observed in the CC category, suggesting that the detected transcriptional changes are primarily associated with functional signaling processes rather than specific cellular compartments.

### 2.5 Transcriptomic signatures associated with survival

As an exploratory analysis, baseline blood transcriptomes of patients with short-term survival (≤3 months; n=10) and long-term survival (>24 months; n=11) were compared. Differential expression analysis of 15,139 (**Supplementary Table 5**) genes identified 177 (148 protein-coding, 17 long non-coding RNA) and 33 (24 protein-coding, 3 long non-coding RNA) differentially expressed genes (DEGs) with padj<0.05, exhibiting log(FoldChange)<-1 and >1, respectively (**Figure 3B**). Among the differentially expressed lncRNAs, several transcripts previously associated with cancer biology and prognosis were identified, including LINC00665, GSEC, MEG3 and SLC12A5-AS1.

**Figure 3:**
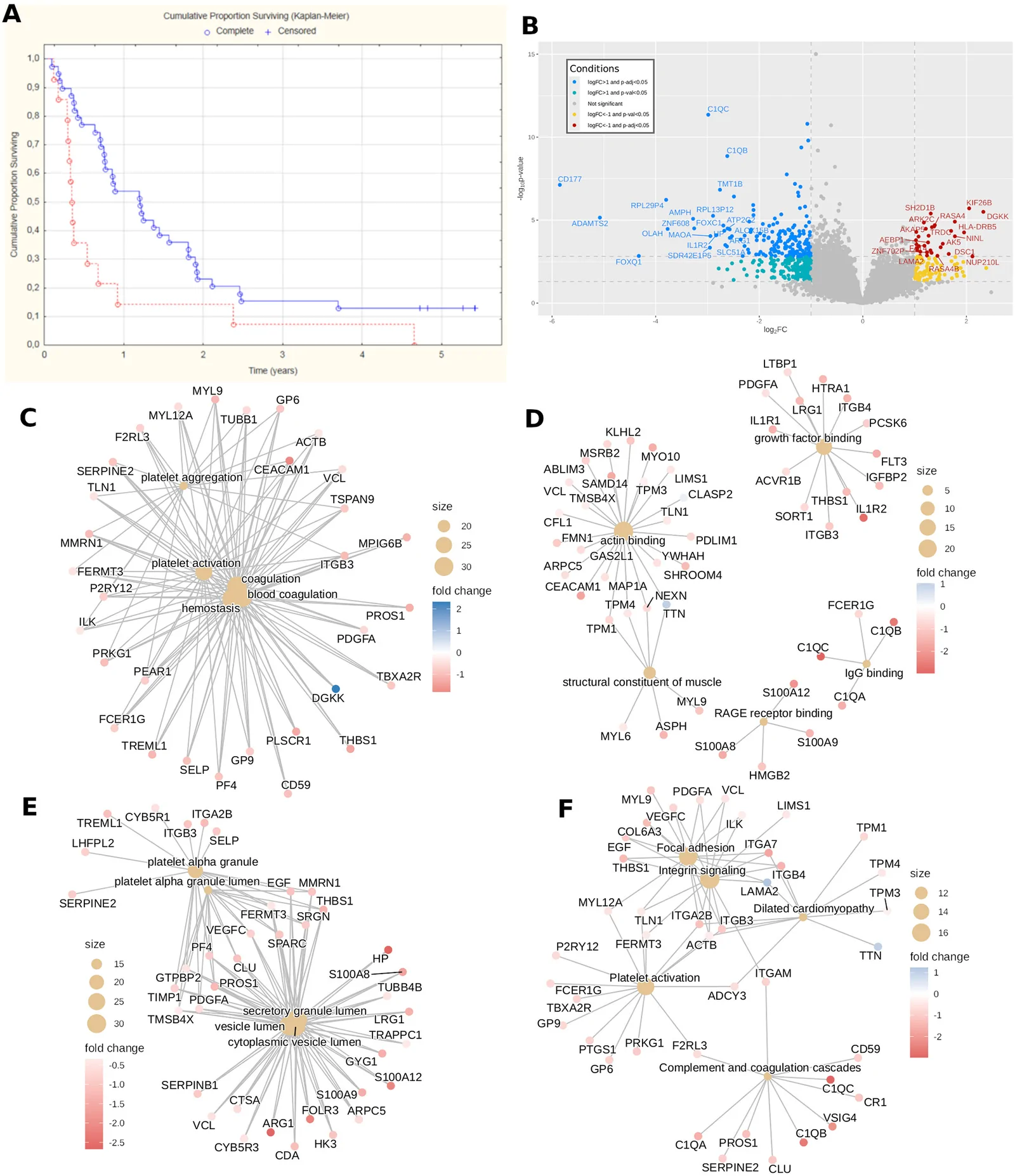
**(A)** Overall survival according to early NT-proBNP increase >135 pg/ml (14 patients) versus no such increase (39 patients); Log-rank test: p<0.03. Cox proportional hazards model: HR = 2.3 (95% CI: 1.22–4.34), Wald test p<0.01 **(B)** Volcano plot showing all analysed genes in the comparison of short- and long-term survival; the top 20 genes by logFC and genes with padj<0.05 and |logFC|>1 are labelled. **(C–F)** Concept network plots showing the top 5 enriched terms: biological process **(C)**, molecular function **(D)**, cellular component **(E)**, and KEGG pathways **(F)**. Node size indicates the number of associated DEGs, and gene colour represents log(FoldChange).

Functional enrichment analysis of DEGs identified 12, 33, and 75 significant GO terms (padj<0.05) according to the MF, CC, and BP aspects, as well as 13 KEGG pathways (**Supplementary Table 5**). The enrichplot of a concept network showed that the most enriched pathways from GO were blood coagulation, hemostasis, and platelet activation (**Figure 3C**), while KEGG analysis highlighted platelet activation, integrin signaling, and focal adhesion pathways (**Figure 3F**). In addition, CC analysis indicated enrichment of genes associated with platelet granules and vesicle-related compartments, including secretory granule lumen, cytoplasmic vesicle lumen, platelet alpha granule, and tertiary granule, as well as cellular structures involved in cell adhesion and migration, such as focal adhesion, cell leading edge, and cell cortex (**Figure 3E**). MF analysis further highlighted functions related to actin binding and actin filament binding, protein heterodimerization, and growth factor and protease binding, suggesting the involvement of cytoskeletal remodeling, cell adhesion, and intercellular signaling processes (**Figure 3D**). Together, these results support the activation of coordinated processes linking platelet function, cytoskeletal dynamics, and cell migration, which may underlie both thromboinflammatory responses and adverse clinical outcomes. To address the potential selection bias associated with the comparison of extreme survival phenotypes, additional Cox proportional hazards regression analyses were performed using the entire study cohort and pathway activity scores as continuous variables. After adjustment for sex and NT-proBNP change, pathways related to focal adhesion (HR = 2.24, 95% CI: 1.24–4.04, FDR = 0.034), regulation of actin cytoskeleton (HR = 2.45, 95% CI: 1.25–4.81, FDR = 0.034) and platelet activation (HR = 2.16, 95% CI: 1.15–4.05, FDR = 0.043) remained significantly associated with overall survival. Similar associations were observed for complement and coagulation cascades (HR = 2.11, 95% CI: 1.09–4.08, FDR = 0.056) and integrin signaling (HR = 1.83, 95% CI: 1.06–3.15, FDR = 0.056), although these did not remain significant after correction for multiple testing.

### 2.6 Cell-type deconvolution analysis

Because the transcriptomic analyses were performed on whole-blood RNA-seq data, we performed additional cell-type deconvolution analyses to assess whether the observed transcriptomic differences could be explained by variations in peripheral blood cell composition. No significant differences in estimated fractions of major immune cell populations, including neutrophils, monocytes, NK cells, B cells, CD8+ T cells and regulatory T cells, were observed between patients with and without NT-proBNP elevation, either at baseline or after the first cycle of ICI treatment (all adjusted p-values >0.05; **Supplementary Figure S2**-**S6**). Similarly, no significant differences in estimated immune-cell composition were detected between checkpoint 1 and checkpoint 2 samples. To further address the potential influence of blood-cell composition, NLR was evaluated before and after immunotherapy. No significant differences in NLR were observed either between patients with and without NT-proBNP elevation or between sampling time points (**Supplementary Figure S7**). In contrast, significant differences in estimated cell-type composition were observed between advanced NSCLC patients and the control group, including B cells (adjusted p <0.0001), regulatory T cells (adjusted p=0.0139) and uncharacterized cells (adjusted p=0.0011).

## 3 Materials and methods

### 3.1 Study populations

This study was conducted in accordance with the ethical standards of the institutional and/or national research committees, and in accordance with the 1964 Helsinki Declaration and its later amendments (or comparable ethical standards) and was approved by Maria Sklodowska-Curie National Research Institute of Oncology Local Bioethics Board (decision no 13/2020). All participants provided informed consent to participate. The inclusion criteria for the study group patients: age>18 years; confirmed histological diagnosis of advanced/metastatic NSCLC with low PD-L1 expression (TPS<50%); no previous use of ICI therapy; availability of pre‐treatment abdominal CT scans, performed no more than 3 months before starting the immunotherapy. Exclusion criteria: current or suspected interstitial lung disease/pneumonitis; significant pulmonary compromise due to ongoing pulmonary illness; uncontrolled or significant cardiovascular disease; clinically significant corneal disease; active, known, or suspected autoimmune disease; diagnosis of immunodeficiency.

In the period from May 2020 to April 2021, a group of 61 patients were qualified for therapy with immune checkpoint inhibitors. Early cardiac assessment through echocardiography and cardiac biomarker evaluation (troponins and NTproBNP) was performed before starting immunotherapy and after a median of 28 days with an interquartile range of 21–35 days. A subgroup of 53 randomly selected patients underwent such a rigorous early cardiac assessment.

The definition proposed by International Cardio-Oncology Society and later adopted by the European Society of Cardiology has been included as criteria of cancer therapy related cardiac dysfunction (CTRCD) (29, 30). Analyzing the echocardiography results, none of the patients experienced severe cardiac dysfunction with a decrease in left ventricular ejection fraction (EF) below 40%, six patients had a decrease in EF by 10 percentage points from the baseline value, but only one patient had a final EF value in the range of 40-49%, the remaining 5 patients had a final EF value above 50%, which did not meet the criteria for the diagnosis of moderate CTRCD. At that time, global longitudinal strain (GLS) had not yet been assessed, so further analyses focused on the assessment of biomarkers. Detailed baseline and follow-up LVEF values for all patients who underwent serial echocardiographic assessment are presented in **Supplementary Figure S8**. No significant differences in LVEF trajectories were observed between patients with and without NT-proBNP elevation. An increase in troponin from the range of values considered normal to values above the upper limit of normal (≥14 ng/L) was noted in 8 patients. In the group of 53 patients, the NT-proBNP concentrations before starting immunotherapy ranged from 4.6 to 2085 pg/mL (a mean value was 460; a median value 230), and after the therapy the concentrations ranged between 5.1 and 3764 pg/mL (a mean value was 526; a median value 297). A decrease in NTproBNP ccurred in 23 patients. However, the NTproBNP concentration - as a marker of heart failure - significantly increased by a value greater than 135 pg/ml (upper quartile of the general changes) occurred in 14 patients out of these 53 patients. This subgroup was considered clinically relevant because four of the fourteen patients experienced subsequent cardiovascular events during follow-up, including two cases of clinically diagnosed cardiotoxicity, one pulmonary embolism, and one episode of symptomatic atrial fibrillation. This subgroup of 14 patients also had a significantly unfavorable overall survival in long term follow-up (**Figure 3A**).

The comparison of clinical characteristics of the patients with and without an increase of NTproBNP>135 pg/ml at the beginning of therapy with immune checkpoint inhibitors due to lung cancer is presented in the **Table 1**. There were no significant differences between subgroups regarding type of immunotherapy, line of treatment in the palliative setting, clinical stage at the beginning of immunotherapy, previous treatment with chemotherapy or radiotherapy on the lung area, history of thoracic intervention and even history of cardiovascular comorbidity. However patients with an early increase of NTproBNP>135pg/ml discontinued immunotherapy significantly often due to any severe adverse events.

**Table 1:** The comparison of clinical characteristics of the patients with and without an increase of NTproBNP>135 pg/ml at the beginning of therapy with immune checkpoint inhibitors due to lung cancer (chi-square test).

Category	14 patients with an increase of NTproBNP >135 pg/ml	39 remaining patients without increase of NTproBNP	p-value
Type of immunotherapy			p=0.88
Niwolumab	7	17	
Pembrolizumab	4	14	
Atezolizumab	3	8	
Line of cancer therapy aspalliative setting			p=0.62
First	4	14	
Second	10	25	
Clinical stage			p=0.53
III A	2	1	
III B	1	5	
III C	0	1	
IV A	2	6	
IV B	9	26	
Previous chemotherapy (palliative or adjuvant setting)	10	28	p=0.98
Previous radiotherapy on lung area	4	14	p=0.62
Previous thoracic surgery	4	7	p=0.4
History of cardiovascular Comorbidities, including:	8	20	p=0.71
Arterial hypertension	5	15	
Ischemic heart disease	3	9	
Atrial fibrillation	2	2	
Severe adverse events as a reason of discontinuation of immunotherapy	8	4	p=0.0003

The control group consisted of 29 patients (17 women and 12 men, aged 52 – 85; median – 70 years) scheduled for surgical removal of lung tumors, in whom histopathological examination of surgical specimens revealed non-squamous NSCLC, squamous NSCLC, and atypical carcinoid tumors in 17, 8, and 4 patients, respectively. Thirteen, 13, and 3 control patients had stage I, II, or III disease, and none of them had undergone neoadjuvant therapy/surgery prior to blood collection for RNA sequencing. The inclusion criteria for the control group patients: age >18 years; confirmed histopathological diagnosis of lung neoplastic disease. Exclusion criteria: uncontrolled or significant cardiovascular disease; active, known, or suspected autoimmune disease; diagnosis of immunodeficiency.

### 3.2 Blood sample processing and RNA sequencing

Peripheral venous blood samples were collected into Tempus™ Blood RNA Tubes (Thermo Fisher Scientific) according to the manufacturer’s instructions to ensure immediate stabilization of intracellular RNA. After collection, samples were thoroughly mixed, stored at -20 °C, and subsequently transferred to -80 °C until RNA isolation. Total RNA was extracted using the mirVana™ PARIS™ Kit (Thermo Fisher Scientific), enabling efficient recovery of high quality RNA from whole blood samples (15, 31). RNA concentration was measured spectrophotometrically, and RNA integrity was assessed using the Agilent Bioanalyzer (Agilent Technologies) with an RNA analysis kit, according to the manufacturer’s protocol. Only samples with a RNA Integrity Number (RIN) greater than 8.0 were considered of sufficient quality and included in downstream analyses. RNA sequencing libraries were prepared using the Illumina TruSeq™ Stranded Total RNA Library Prep Kit with Ribo Zero/TS Flex workflow, incorporating ribosomal RNA and globin mRNA depletion. Library preparation, quality control, and sequencing were performed according to standardized protocols. Paired end sequencing was carried out on an Illumina MiSeq platform with a read length of 2 × 100 bp, generating approximately 25 million clusters (5 Gb) per sample (15). The resulting RNA seq data were used for transcriptomic analyses of peripheral blood cells.

Sequenced reads were mapped to the human transcriptome from Ensembl (GRCh38, version 115) using the STAR method (version:2.7.10a) (32, 33). Mapped reads were then trimmed with fastp (version:0.20.1) and quantified against transcripts from the GRCh38 database (Ensembl, version 115) using HTSeq count (version:1.99.2) with default parameters, except that the stranded option was set to reverse (34). Genes were trimmed with filterByExpr function from edgeR package (version:4.8.2) (35). Differentially expressed genes (DEGs) were identified with DESeq2, including sex as a covariate in all analyses (36). In the analysis of the early response to ICI, patient ID was additionally included as a covariate to account for inter individual variability. Protein attributes, including protein biotype, were assigned using the AnnotationHub (version 4.0.0) and ensembldb (version 2.34.0) R packages (37, 38), based on Ensembl GRCh38 annotations (release 113).

To assess global variability and potential batch effects, principal component analysis (PCA) was performed on normalized gene expression data using variance-stabilizing transformation. PCA plots were visually inspected to evaluate sample clustering patterns and to identify potential outliers or technical biases. P values for differentially expressed genes were adjusted for multiple testing using the Benjamini–Hochberg procedure, and genes with adjusted p values padj<0.05 were considered significant. Functional overrepresentation analysis based on Gene Ontology (GO) and KEGG databases was performed using the enrichGO and enrichKEGG functions from clusterProfiler (version:4.18.4) (39, 40). Default settings were applied, with padj-values corrected using the Benjamini–Hochberg method and a significance threshold of padj<0.05. Data visualisation was carried out using ggplot2 (version: 4.0.2), patchwork (version:1.3.2) and enrichplot (version: 1.30.5) (41–43).

To investigate whether transcriptomic differences could be influenced by variations in peripheral blood-cell composition, cell-type deconvolution analysis was performed on normalized whole-blood RNA-seq data using quanTIseq from R package immunedeconv (version: 2.1.4) (44, 45). In addition, neutrophil-to-lymphocyte ratio (NLR) was calculated using absolute neutrophil and lymphocyte counts obtained from routine blood tests. NLR values were compared between patients with and without NT-proBNP elevation and between sampling time points before and after immunotherapy using the Mann-Whitney U test. Statistical analyses and graphical visualization were performed using GraphPad Prism.

Ethical approval declarations This study was approved by the Local Bioethics Committee of the Maria Sklodowska-Curie National Research Institute of Oncology, Warsaw, Poland (approval no. 13/2020). The study was conducted in accordance with the principles of the Declaration of Helsinki and its subsequent amendments. Written informed consent was obtained from all participants prior to enrollment.

## 4 Discussion

Anticancer therapies, including anthracyclines, fluoropyrimidines, taxanes, tyrosine kinase inhibitors, and immunotherapies, can damage DNA and activate proinflammatory and proapoptotic signaling pathways in cardiomyocytes and endothelial cells, causing cardiovascular toxicity (46). The degree of toxicity varies depending on the treatment modality and may have different etiologies. Immunotherapy-induced cardiotoxicity, induced ICIs, results from T-cell infiltration, and manifests as myocarditis and acute cardiac injury, leading to potentially fatal cardiovascular complications, with mortality rates reaching 50% (47). Late cardiotoxicity is a major consequence of radiotherapy, with an estimated incidence of >10% (48–50).

Cardiotoxicity associated with ICI treatment is considered a major cause of long-term morbidity and mortality among cancer survivors (51, 52). Although studies suggest that baseline troponin levels may be useful in predicting cardiotoxicity (53–55), there is no consensus on standard monitoring protocols for diagnosing ICI-induced cardiotoxicity (56). In this study, we investigated blood transcriptomic signatures associated with susceptibility to and early molecular events linked to an ICI-related cardiac biomarker–based phenotype in patients with NSCLC. Previous studies have demonstrated that blood-derived transcriptomic profiles, including analyses performed in PBMCs, can distinguish between controls and patients with a wide range of autoimmune, inflammatory, infectious, cardiovascular and neoplastic disorders, as well as responses to environmental stressors (57).

We observed baseline transcriptional differences in patients who subsequently developed a cardiac biomarker-based phenotype, which may indicate the presence of pre-existing alterations in regulatory and signaling pathways prior to treatment initiation. Furthermore, early transcriptional changes following ICI therapy were observed exclusively in this subgroup, potentially reflecting activation of immune-related processes. These findings may suggest that ICI-related cardiovascular toxicity, as reflected by NT-proBNP-defined changes, may arise in the context of a pre-existing state of immune dysregulation observed in our cohort, which upon treatment may lead to a distinct molecular response.

The baseline differences identified in this study point towards subtle but coordinated alterations in regulatory and signaling pathways in patients predisposed to the development of a cardiac biomarker-defined phenotype. Among the deregulated genes, several members of the zinc finger (ZNF) family may reflect changes in transcriptional regulation, possibly indicating a primed immune or stress-responsive state (58). Notably, the upregulation of DACT1, a component of the WNT signaling pathway, may suggest involvement of pathways previously associated with immune modulation, inflammation, and tissue remodeling (59). In contrast, the downregulation of genes such as MMP1 and OXTR may reflect alterations in extracellular matrix remodeling and neuroendocrine signaling (60). Reduced expression of the oxytocin receptor is of particular interest, given its reported role in modulating inflammation and cardiovascular function, and previous studies suggesting anti-inflammatory and cardioprotective effects of oxytocin signaling (61). This may indicate a potential link between impaired protective signaling and increased vulnerability to cardiovascular biomarker alterations during ICI therapy. However, given the limited number of differentially expressed genes, these interpretations should be considered exploratory and require validation in larger cohorts.

A key observation of this study is that early transcriptional changes following ICI administration were present only in patients who developed a cardiac biomarker-defined phenotype. Although these changes were modest in magnitude and limited in number, they were consistent and may reflect activation of innate inflammatory and immune-related processes. These findings may point to early activation of inflammatory signaling pathways, which could contribute to immune-mediated tissue damage (62). The absence of comparable changes in patients without the NT-proBNP-defined cardiovascular biomarker phenotype may indicate a more specific biological response, rather than a general effect of ICI therapy (63, 64). Given the limited number of differentially expressed genes, these findings should be interpreted as a preliminary signal rather than a robust transcriptional response.

The comparison between patients with and without a cardiac biomarker-defined phenotype suggests a potential involvement of adaptive immune mechanisms, as indicated by enrichment of pathways related to T-cell activation and differentiation. The observed transcriptional patterns may indicate enhanced T-cell-mediated immune activation, suggesting that dysregulated adaptive responses could contribute to the development of cardiotoxicity (64, 65). To address the potential influence of blood-cell composition on transcriptomic profiles, we additionally performed cell-type deconvolution analysis and assessed NLR. Importantly, no significant differences in estimated proportions of major immune-cell populations or NLR values were observed between patients with and without NT-proBNP elevation, either before or after ICI treatment. Therefore, the observed transcriptomic signatures are unlikely to be explained solely by shifts in circulating immune-cell composition, although residual compositional effects inherent to whole-blood transcriptomic analyses cannot be completely excluded. Although cell-type deconvolution analysis and NLR assessment did not reveal significant differences in major immune-cell populations between groups, the precise cellular origin of the identified transcriptomic signatures could not be directly resolved using bulk whole-blood RNA-seq data. Future studies integrating bulk transcriptomic profiles with single-cell RNA-seq datasets may provide additional mechanistic insight into the immune-cell populations contributing to these associations.

These observations are in line with the known mechanism of action of ICIs, which enhance T-cell activity but may also lead to off-target immune-related adverse events. The identified transcriptomic signatures may therefore reflect an exaggerated or dysregulated immune response contributing to cardiovascular complications (62, 66).

Transcriptomic differences associated with survival revealed enrichment of pathways related to coagulation, platelet activation, and integrin signaling. In our cohort, these pathways may reflect a link between thromboinflammatory processes and adverse clinical outcomes (66). The overlap between coagulation-related pathways and adverse clinical outcomes may suggest that thromboinflammatory mechanisms contribute both to disease progression and to treatment-related complications. Importantly, the interpretation of coagulation- and platelet-related transcriptomic signatures was not supported by independent laboratory markers of coagulation activation, such as D-dimer or fibrinogen levels, which were not available in the present study. Consequently, the observed thromboinflammatory patterns should be considered preliminary; they require further validation in future studies. Importantly, the association between coagulation-, platelet activation- and adhesion-related pathways and overall survival was additionally evaluated using Cox proportional hazards regression models including the entire study cohort. Similar pathway groups remained associated with overall survival after adjustment for sex and changes in NT-proBNP levels, providing additional support for these observations. Nevertheless, because only all-cause mortality data were available, these findings should not be interpreted as evidence of a specific relationship with cardiovascular mortality. In particular, the survival analysis was based on a highly selected subgroup of only 21 patients representing extreme survival phenotypes and should therefore be considered exploratory and hypothesis-generating rather than confirmatory (67, 68). Moreover, multiple transcriptomic comparisons were performed within the same study cohort. Although false-discovery-rate correction was applied within each analysis, the results should be interpreted in the context of multiple exploratory evaluations addressing different biological questions.

Finally, global transcriptomic differences between advanced and early-stage NSCLC patients primarily reflected metabolic and proliferative processes, including cellular respiration, cell cycle progression, and protein activity. These findings are consistent with the increased metabolic demands and systemic alterations associated with advanced malignancy and should be interpreted as a background signal rather than a direct mechanism of cardiotoxicity (69). Importantly, these differences may also reflect the confounding effects of disease stage and prior anticancer treatments, which were not evenly distributed between groups.

A substantial proportion of differentially expressed transcripts identified across several comparisons corresponded to lncRNAs. Among them were transcripts previously implicated in immune regulation, inflammation, cancer progression and prognosis, including TLR8-AS1, GAS6-AS1, LINC00861 and MEG3, as well as immune-related lncRNAs identified in the NT-proBNP-defined phenotype analysis, such as MAILR (macrophage interferon regulatory lncRNA) and MIR181A1HG (70–73). In the survival-associated comparison, differential expression of lncRNAs including LINC00665, GSEC, MEG3 and SLC12A5-AS1 was observed, suggesting that non-coding regulatory mechanisms may also contribute to transcriptomic signatures associated with disease progression and clinical outcome (74–76). However, functional characterization of many lncRNAs remains incomplete, and their biological significance in the context of immune checkpoint inhibitor therapy requires further investigation. Consistent with previous observations regarding the structure of KEGG annotations, several neurodegenerative disease- and infection-related pathways initially identified in the enrichment analysis reflected broad overlap of genes involved in oxidative phosphorylation and general inflammatory or stress-response processes. These pathways were therefore not interpreted as disease-specific biological mechanisms and were excluded from the final visualization to avoid overinterpretation.

The relatively small sample size, particularly in subgroup analyses (e.g., survival and cardiotoxicity groups), represents a limitation of this study and may reduce statistical power and increase the risk of false-positive findings. Another important limitation is the use of NT-proBNP levels as a surrogate marker of cardiotoxicity. Although NT-proBNP is widely used in clinical practice as a biomarker of cardiac dysfunction, it does not represent a direct diagnosis of ICI-related cardiotoxicity and may be influenced by other factors such as age, renal function, or comorbidities (28, 77). Importantly, NT-proBNP elevation should not be interpreted as equivalent to clinically confirmed ICI-related cardiotoxicity. NT-proBNP may reflect a range of cardiovascular and systemic conditions, including volume overload, atrial arrhythmias, renal dysfunction and other comorbidities. Therefore, the present study should be interpreted as an investigation of an NT-proBNP-defined cardiovascular biomarker phenotype rather than a direct analysis of confirmed ICI-related cardiotoxicity. Additionally, this marker does not distinguish between toxic effects of ICI therapy and chronic toxicity associated with previously administered therapies. In fact, blood transcriptomic profiling reflects systemic immune responses and may not fully capture tissue-specific processes occurring in the myocardium, which limits direct mechanistic interpretation (78). The findings were not validated in an independent external cohort, which limits their generalizability and requires further confirmation. In addition, reliable cause-of-death information was unavailable for the majority of deceased patients. Consequently, competing-risk analyses distinguishing cardiovascular mortality from cancer-related mortality could not be performed, and all survival analyses reported in this study refer to all-cause mortality. Due to the observational nature of the study, the identified associations do not imply causality between baseline transcriptional signatures and cardiotoxicity development. Therefore, these differences should be interpreted with caution, as they may primarily reflect disease stage and/or and consequences of previous anticancer treatment rather than mechanisms directly related to cardiotoxicity. While paired analyses allowed for partial control of inter-individual variability, other comparisons were based on cross-sectional designs, which may be influenced by patient-specific factors not fully captured in the model. Additionally, the observed transcriptomic patterns may be influenced by inter-individual variability and clinical heterogeneity, which could not be fully accounted for despite covariate adjustment.

## 5 Conclusion

This study provides initial evidence that blood transcriptomic profiling may reflect both baseline differences associated with susceptibility and early molecular responses related to ICI-related cardiovascular alterations, as reflected by NT-proBNP-defined changes in this cohort of NSCLC patients. The identification of pre-treatment differences, together with treatment-induced transcriptional changes observed in patients developing a cardiac biomarker-defined phenotype, is consistent with the presence of an underlying predisposed biological state. Although further validation in larger and independent cohorts is required, these findings highlight the potential of peripheral blood transcriptomic profiling as a minimally invasive approach to provide insight into the biological mechanisms underlying immune-related cardiovascular complications. Such insights may contribute to the development of improved risk stratification and more personalized monitoring strategies in patients undergoing ICI therapy.

## Data Availability

The RNA sequencing datasets generated and/or analyzed during the current study are available in the SRA repository, accession number PRJNA1490626. Code to the statistiacal analyses is available in repository: https://doi.org/10.5281/zenodo.21224402.

## Supplementary material

Supplementary Table 1 Data about genes and enrichment Gene Ontologies from analyses global transcriptomic differences between advanced and early-stage NSCLC (XLSX).

Supplementary Table 2 Data about genes and enrichment Gene Ontologies from analyses baseline transcriptomic signatures associated with cardiotoxicity susceptibility (XLSX).

Supplementary Table 3 Data about genes and enrichment Gene Ontologies from analyses early response to ICI (XLSX).

Supplementary Table 4 Data about genes and enrichment Gene Ontologies from analyses transcriptomic signatures associated with cardiac biomarker-defined phenotype (XLSX).

Supplementary Table 5 Data about genes and enrichment Gene Ontologies from analyses transcriptomic signatures associated with survival (XLSX).
