# Supplementary material for: Cardiovascular biomarker phenotype associated with immune checkpoint inhibitor therapy in patients with non-small cell lung cancer: an exploratory blood transcriptome study
Source: Front Immunol. 2026 Oct 21;17:1972440. doi: 10.3389/fimmu.2026.1972440 (PMC13733417; doi:10.3389/fimmu.2026.1972440)
Supplement: Supplementary file 1 [file DataSheet1.pdf]

Comparison of estimated immune-cell composition and neutrophil-tolymphocyte ratio (NLR) between study groups and sampling time points.

1. Global transcriptomic differences between and early-stage NSCLC

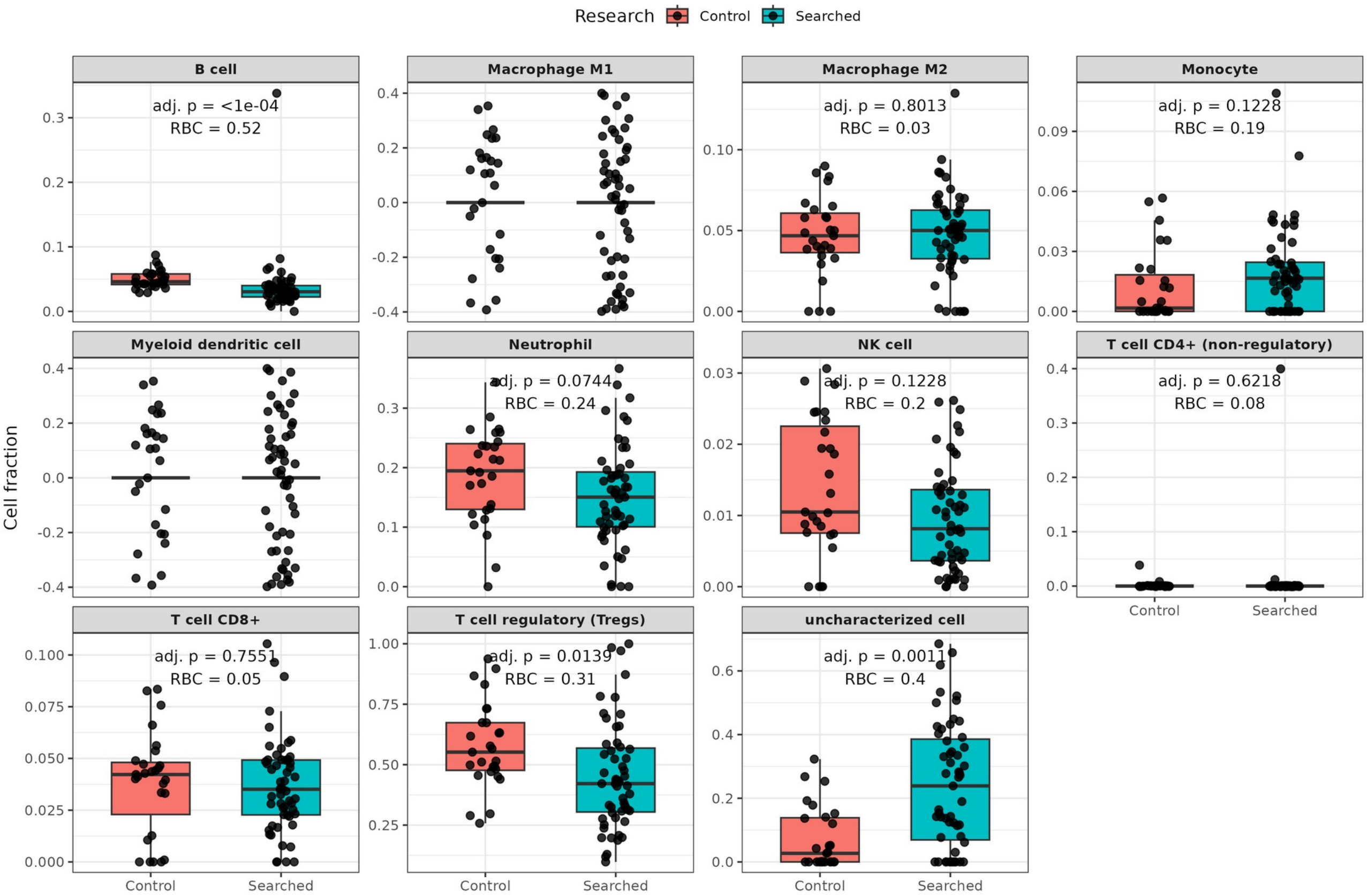

Figure S2. Differences in immune cell fractions estimated by transcriptome deconvolution between early-stage NSCLC (Control) and advanced NSCLC (Searched) groups. The relative fractions of immune cell populations were estimated from whole-blood transcriptome profiles using transcriptome-based immune cell deconvolution. Boxplots show the distribution of estimated cell fractions in the Control and Searched groups, with individual points representing individual samples. Differences between groups were assessed statistically, and adjusted *p*-values (adj.p) are shown in each panel. The rank-biserial correlation (RBC) is provided as a measure of the effect size. The analysis included B cells, macrophage M1 and M2 populations, monocytes, myeloid dendritic cells, neutrophils, NK cells, non-regulatory CD4+ T cells, CD8+ T cells, regulatory T cells (Tregs), and uncharacterized cells. The analysis indicated significant differences (adjusted $p < 0.05$) in the proportions of B cells (adjusted $p = 0.0004$, RBC = 0.52) and regulatory T cells (adjusted $p = 0.0139$, RBC = 0.31) between the control and searched groups.

2. Baseline transcriptomic signatures associated with cardiotoxicity susceptibility

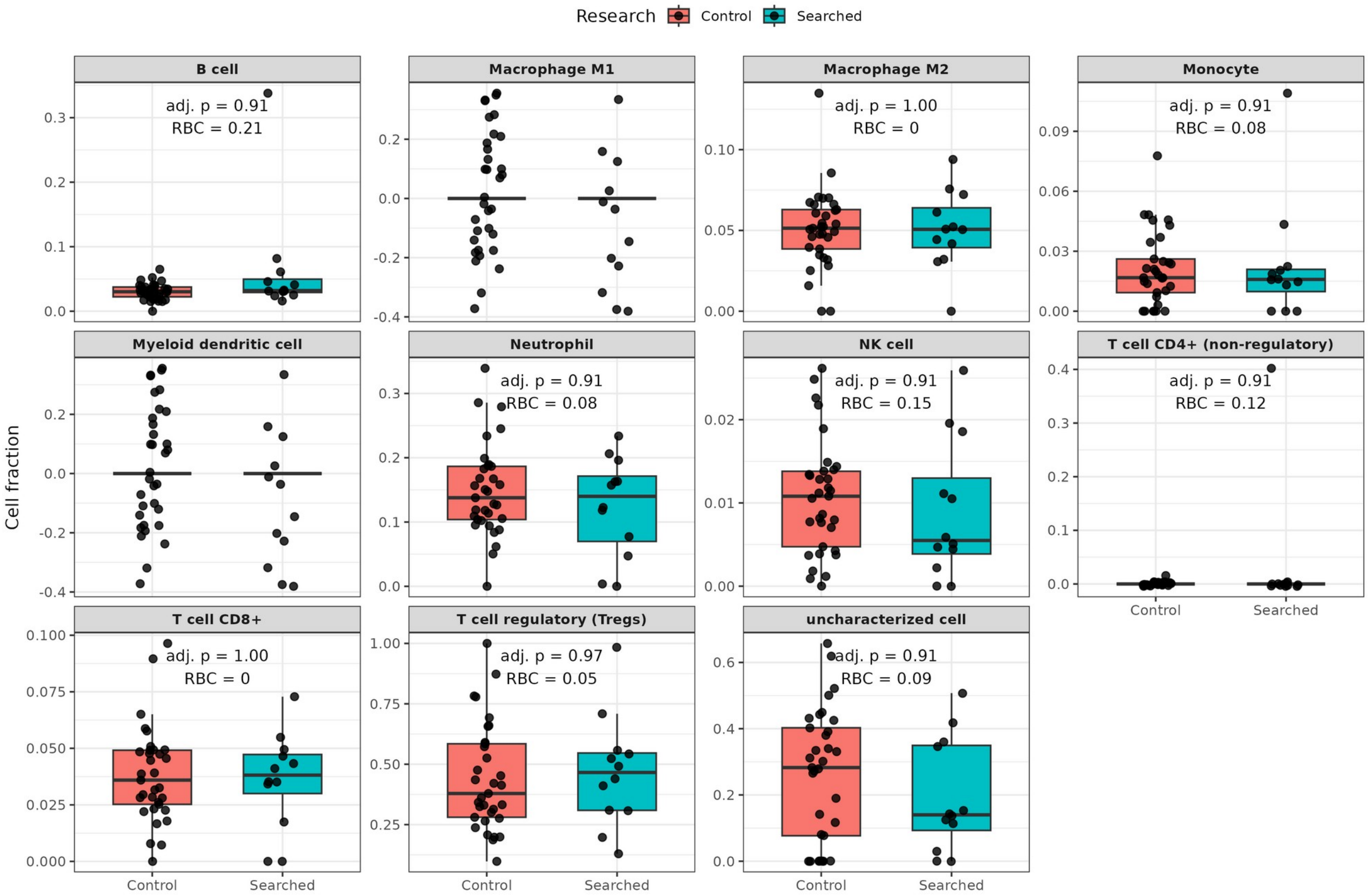

Figure S3. Differences in estimated immune cell fractions between the Control and cardiotoxicity-susceptible (Searched) groups based on transcriptome deconvolution. The relative fractions of immune cell populations were estimated from whole-blood transcriptome profiles using transcriptome-based immune cell deconvolution. Boxplots show the distribution of estimated cell fractions in the Control and Searched groups, with individual points representing individual samples. Differences between groups were assessed statistically, and adjusted *p*-values (adj.p) are shown in each panel. The rank-biserial correlation (RBC) is provided as a measure of the effect size. The analysis included B cells, macrophage M1 and M2 populations, monocytes, myeloid dendritic cells, neutrophils, NK cells, non-regulatory CD4+ T cells, CD8+ T cells, regulatory T cells (Tregs), and uncharacterized cells. The analysis did not indicate any significant differences (adjusted $p < 0.05$) in the proportions of the analyzed cell types between the groups.

3. Early response to ICI

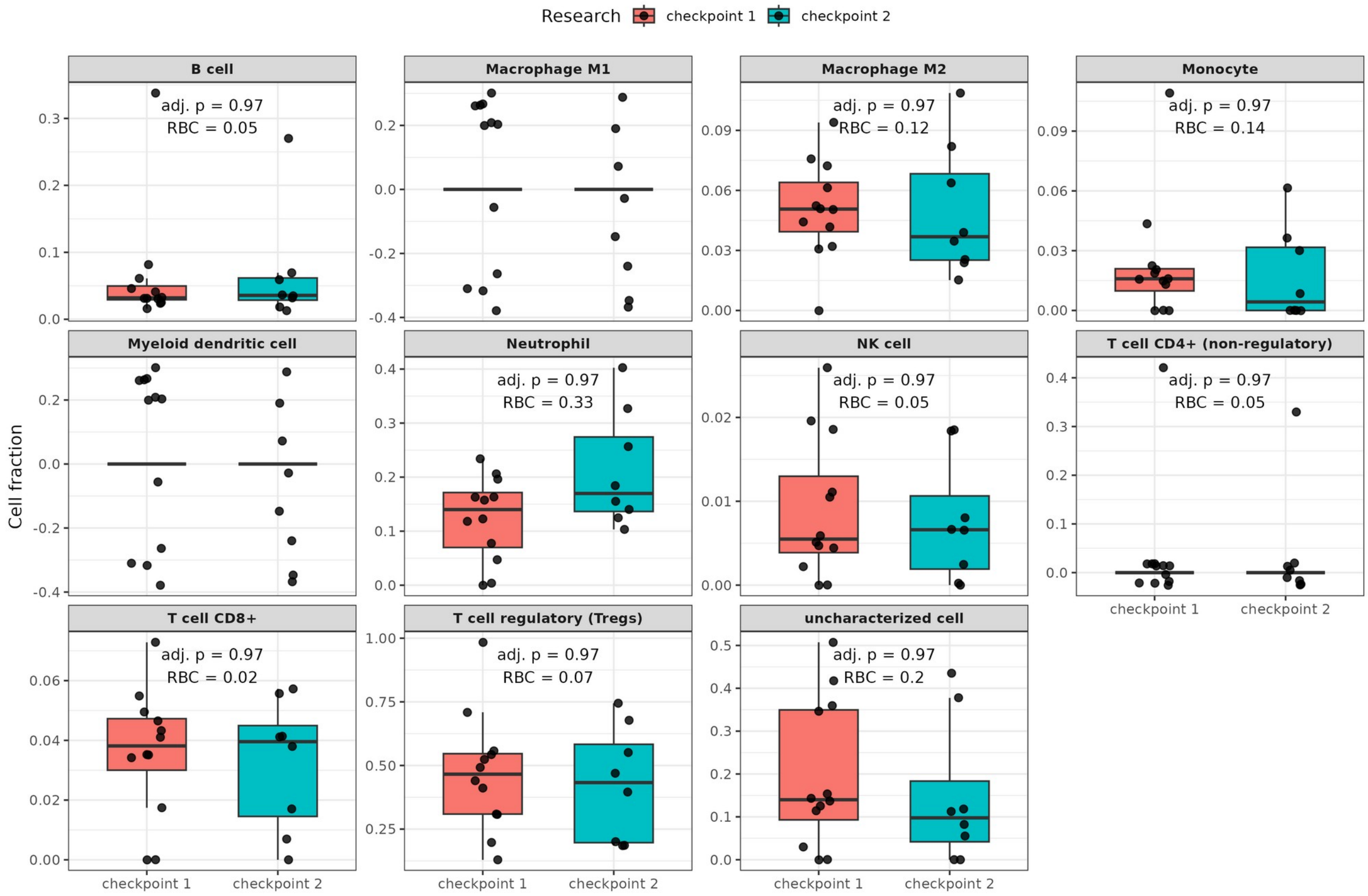

Figure S4. Differences in immune cell fractions estimated by transcriptome deconvolution in patients with cardiotoxicity before (Control) and after (Searched) ICI treatment. The relative fractions of immune cell populations were estimated from whole-blood transcriptome profiles using transcriptome-based immune cell deconvolution. Boxplots show the distribution of estimated cell fractions in the Control and Searched groups, with individual points representing individual samples. Differences between groups were assessed statistically, and adjusted *p*-values (adj.p) are shown in each panel. The rank-biserial correlation (RBC) is provided as a measure of the effect size. The analysis included B cells, macrophage M1 and M2 populations, monocytes, myeloid dendritic cells, neutrophils, NK cells, non-regulatory CD4+ T cells, CD8+ T cells, regulatory T cells (Tregs), and uncharacterized cells. The analysis did not indicate any significant differences (adjusted $p < 0.05$) in the proportions of the analyzed cell types between the groups.

4. Transcriptomic signatures associated with cardiac biomarker-defined phenotype

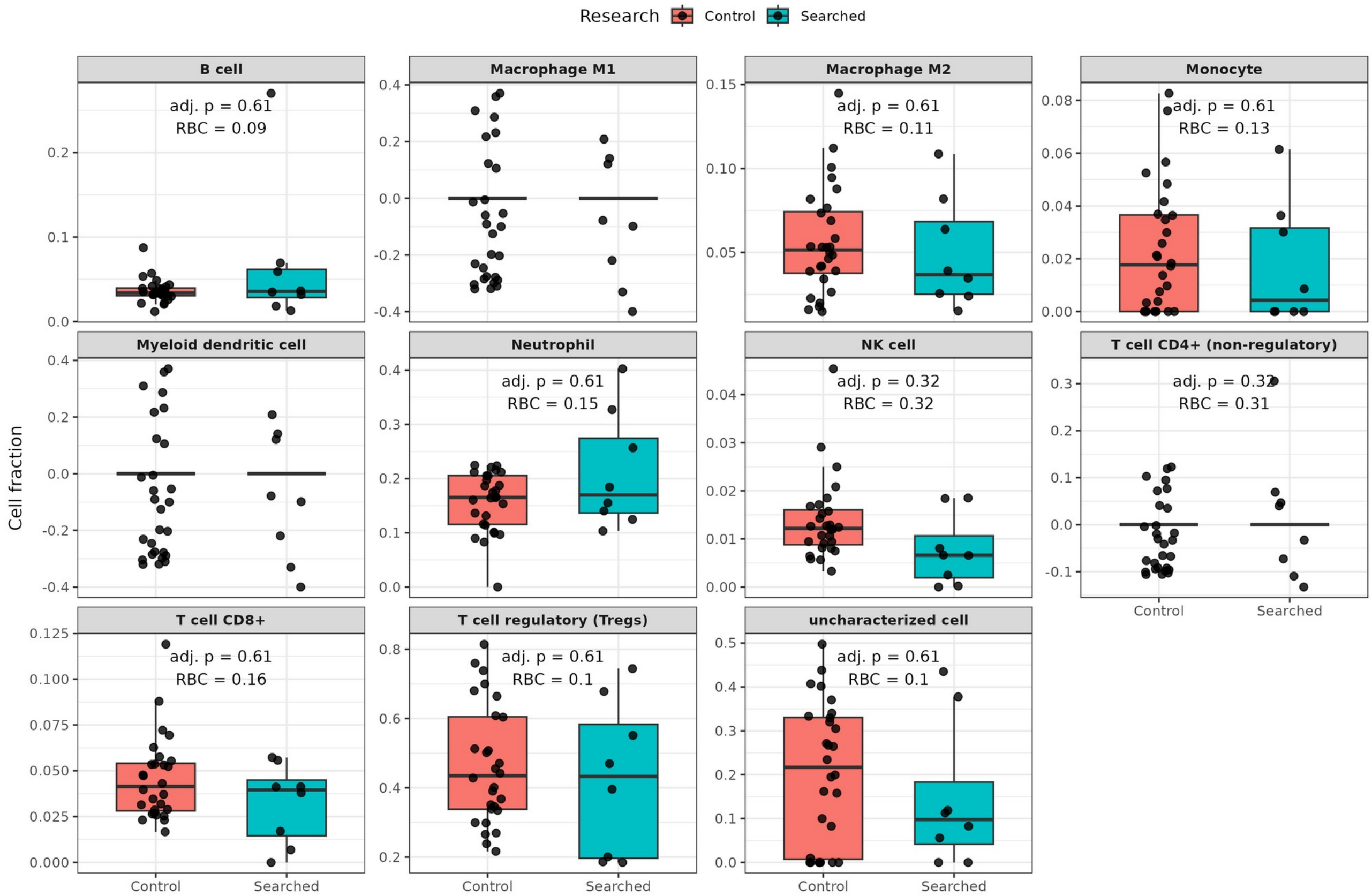

Figure S5. Differences in immune cell fractions estimated by transcriptome deconvolution after the first cycle of ICI treatment between patients without cardiotoxicity (Control) and patients with cardiotoxicity (Searched). The relative fractions of immune cell populations were estimated from whole-blood transcriptome profiles using transcriptome-based immune cell deconvolution. Boxplots show the distribution of estimated cell fractions in the Control and Searched groups, with individual points representing individual samples. Differences between groups were assessed statistically, and adjusted *p*-values (adj.p) are shown in each panel. The rank-biserial correlation (RBC) is provided as a measure of the effect size. The analysis included B cells, macrophage M1 and M2 populations, monocytes, myeloid dendritic cells, neutrophils, NK cells, non-regulatory CD4+ T cells, CD8+ T cells, regulatory T cells (Tregs), and uncharacterized cells. The analysis did not indicate any significant differences (adjusted $p < 0.05$) in the proportions of the analyzed cell types between the groups.

5. Transcriptomic signatures associated with survival

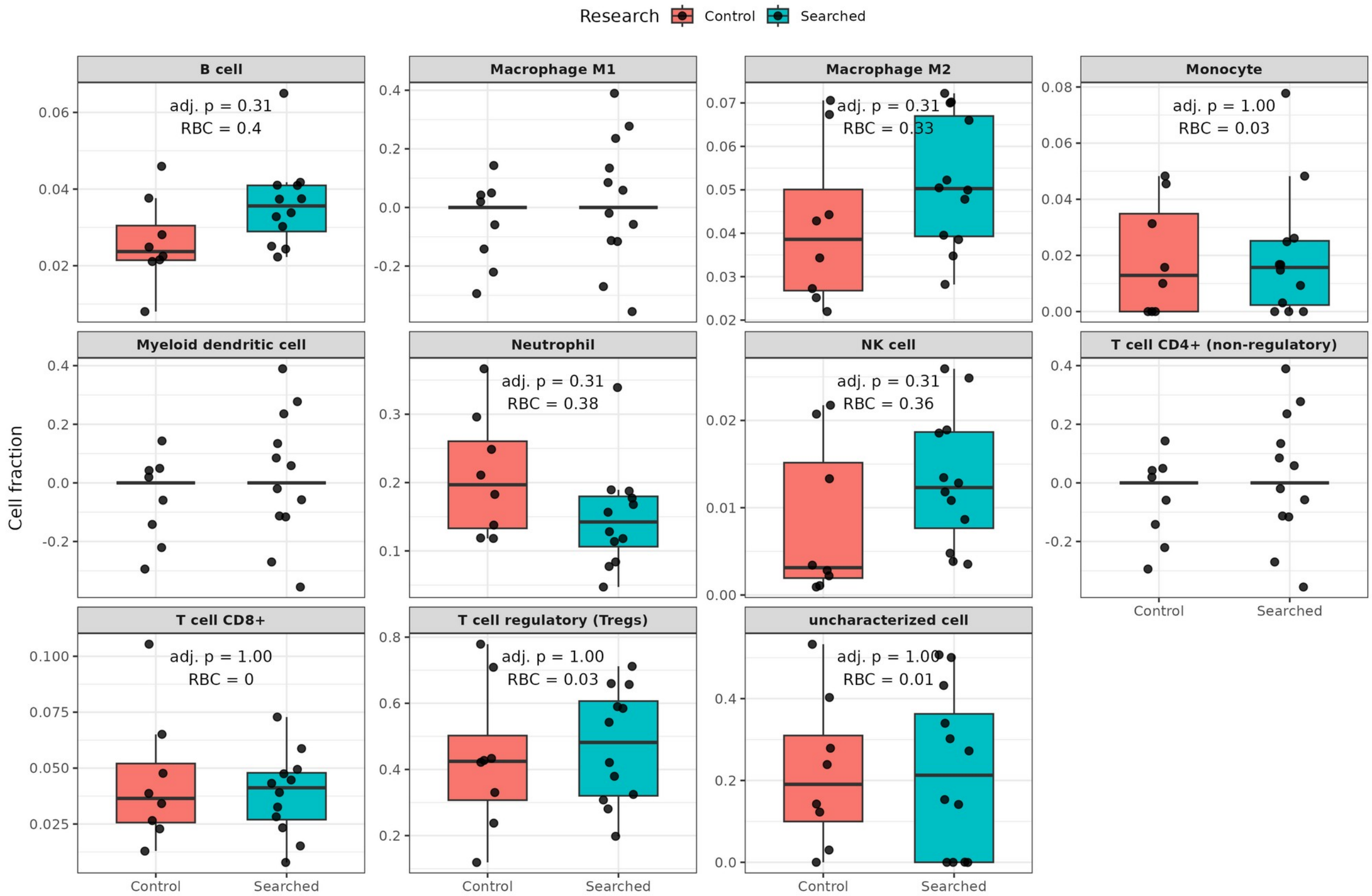

Figure S6. Differences in immune cell fractions estimated by transcriptome deconvolution between patients with short-term (Control) and long-term (Searched) survival. The relative fractions of immune cell populations were estimated from whole-blood transcriptome profiles using transcriptome-based immune cell deconvolution. Boxplots show the distribution of estimated cell fractions in the Control and Searched groups, with individual points representing individual samples. Differences between groups were assessed statistically, and adjusted *p*-values (adj.p) are shown in each panel. The rank-biserial correlation (RBC) is provided as a measure of the effect size. The analysis included B cells, macrophage M1 and M2 populations, monocytes, myeloid dendritic cells, neutrophils, NK cells, non-regulatory CD4+ T cells, CD8+ T cells, regulatory T cells (Tregs), and uncharacterized cells. The analysis did not indicate any significant differences (adjusted $p < 0.05$) in the proportions of the analyzed cell types between the groups.

6. Cell-type deconvolution analysis

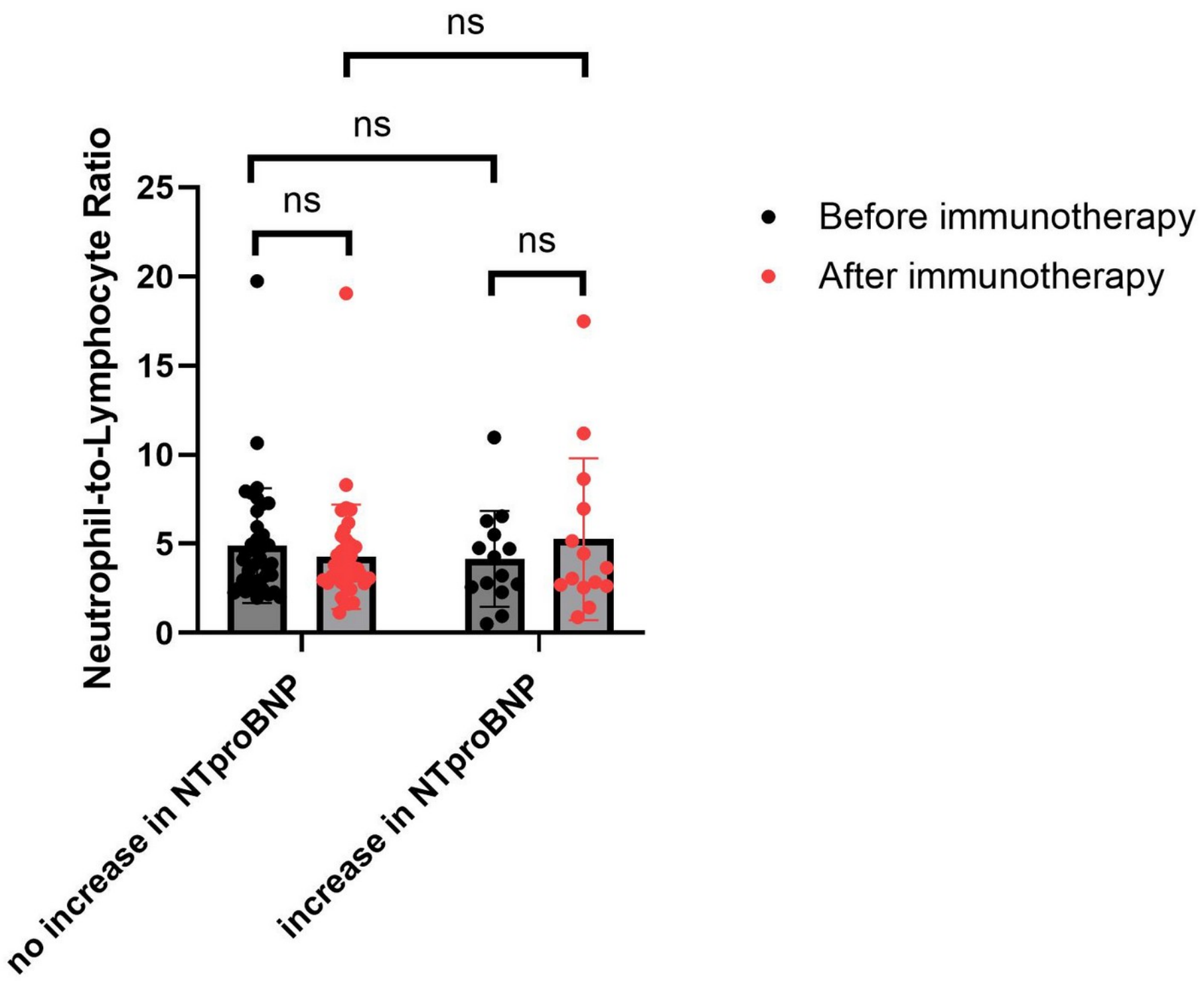

Figure S7. Neutrophil-to-lymphocyte ratio in groups without and with an increase in NT-proBNP >135 pg/mL. Boxplots show the distribution of the neutrophil-to-lymphocyte ratio in the two groups. Red dots indicate measurements obtained before immunotherapy, whereas black dots indicate measurements obtained after immunotherapy. The Wilcoxon test did not indicate statistically significant differences between the groups.
