# Supplementary material for: Cardiovascular biomarker phenotype associated with immune checkpoint inhibitor therapy in patients with non-small cell lung cancer: an exploratory blood transcriptome study
Source: Front Immunol. 2026 Oct 21;17:1972440. doi: 10.3389/fimmu.2026.1972440 (PMC13733417; doi:10.3389/fimmu.2026.1972440)
Supplement: Supplementary file 2 [file DataSheet2.pdf]

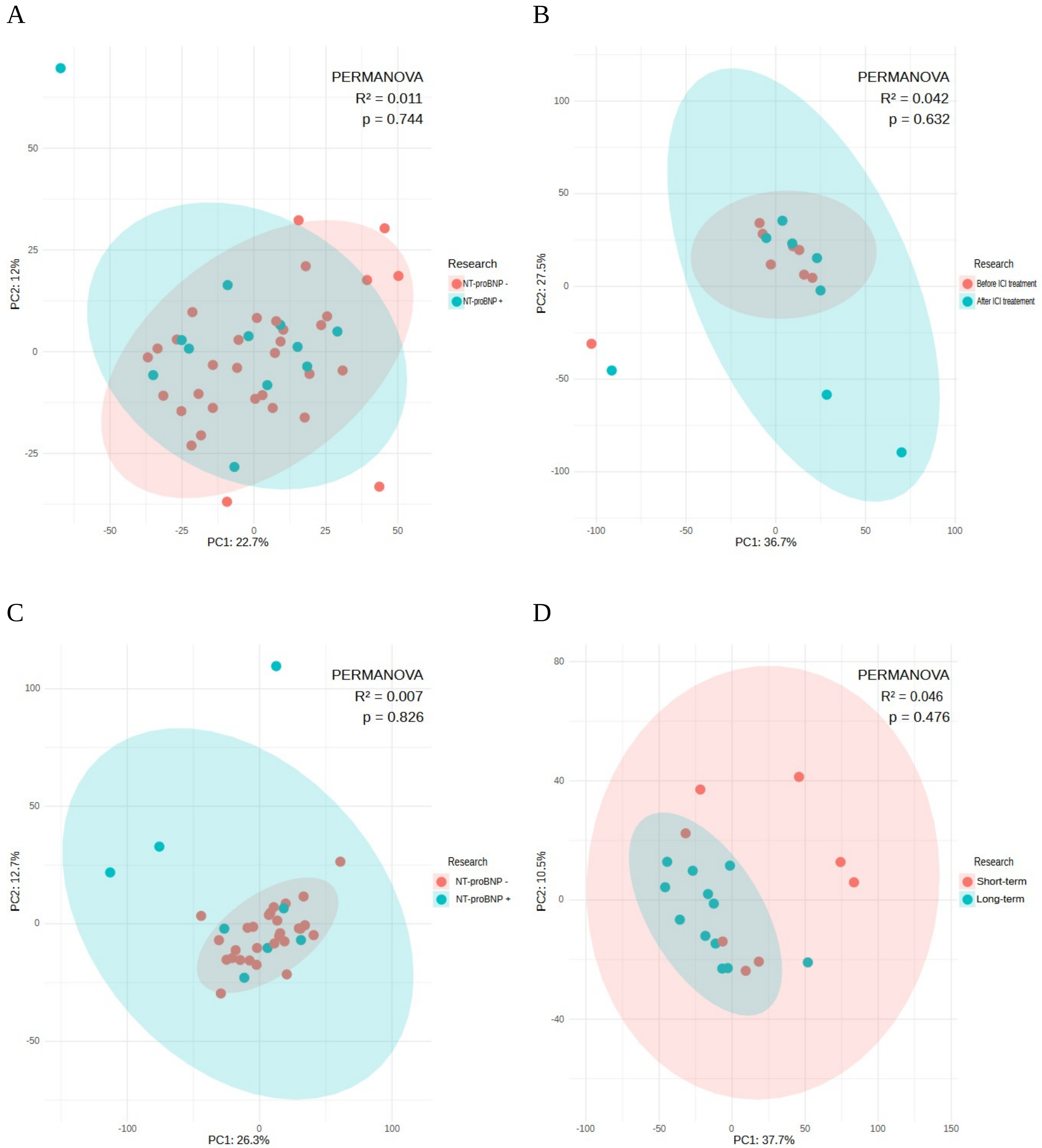

Figure S1. Principal Component Analysis (PCA) of four pairs of datasets. Blue dots represent the study group, while red dots represent the control groups. Ellipses indicate 95% confidence intervals for each group. Panel A shows probes used to analyze signatures associated with cardiotoxicity susceptibility. Panel B corresponds to early response to immune checkpoint inhibitor (ICI) therapy. Panel C contains data used to analyze signatures associated with cardiac biomarker-defined phenotypes. Panel D shows transcriptomic signatures associated with survival.
