# Supplementary material for: Cardiovascular biomarker phenotype associated with immune checkpoint inhibitor therapy in patients with non-small cell lung cancer: an exploratory blood transcriptome study
Source: Front Immunol. 2026 Oct 21;17:1972440. doi: 10.3389/fimmu.2026.1972440 (PMC13733417; doi:10.3389/fimmu.2026.1972440)
Supplement: Supplementary file 3 [file DataSheet3.pdf]

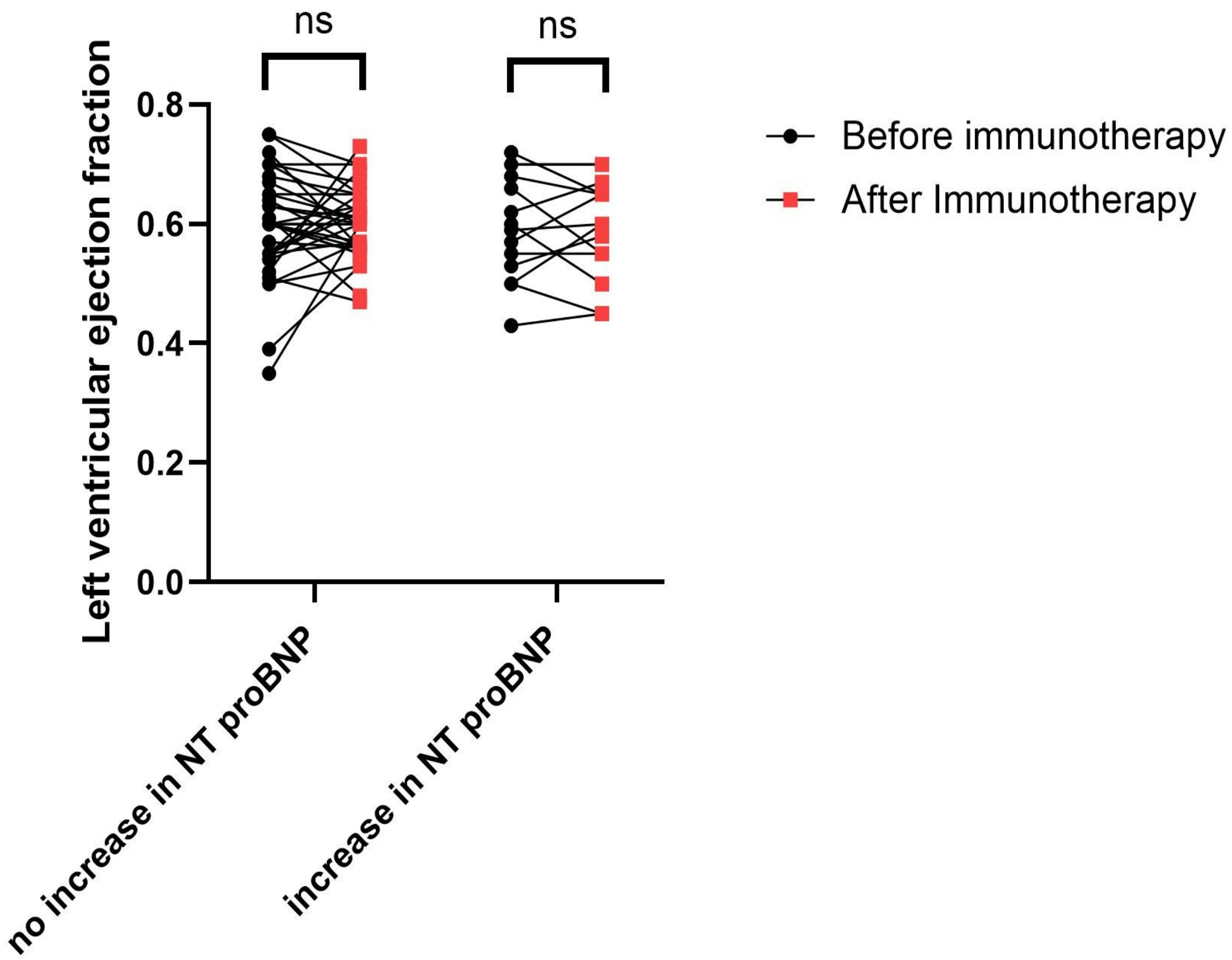

Figure S8. Individual changes in left ventricular ejection fraction (LVEF) during immunotherapy. Patients were divided into two groups without and with increase in NT-proBNP>135 pg/ml after immunotherapy. Black circles represent individual LVEF measurements obtained before immunotherapy and red squares represent measurements obtained after immunotherapy. Lines connect paired measurements from the same patient and illustrate individual changes in LVEF during treatment. No statistically significant differences in LVEF between measurements obtained before and after immunotherapy were observed in either groups (ns).
